# Investigation of predictors of severity of diabetes complications among hospitalized patients with diabetes in Florida, 2016–2019

**DOI:** 10.1186/s12889-023-17288-x

**Published:** 2023-12-05

**Authors:** Jennifer Lord, Keshia Reid, Chris Duclos, Alan Mai, Agricola Odoi

**Affiliations:** 1 Department of Biomedical and Diagnostic Sciences, College of Veterinary Medicine, The University of Tennessee, Knoxville, TN USA; 2 Florida Department of Health, Tallahassee, FL USA

**Keywords:** Diabetes, Complications, Florida, Adapted Diabetes Complications Severity Index scores, aDCSI scores, Generalized estimating equations, GEE, Epidemiology

## Abstract

**Background:**

Severe diabetes complications impact the quality of life of patients and may lead to premature deaths. However, these complications are preventable through proper glycemic control and management of risk factors. Understanding the risk factors of complications is important in guiding efforts to manage diabetes and reduce risks of its complications. Therefore, the objective of this study was to identify risk factors of severe diabetes complications among adult hospitalized patients with diabetes in Florida.

**Methods:**

Hospital discharge data from 2016 to 2019 were obtained from the Florida Agency for Health Care Administration through a Data Use Agreement with the Florida Department of Health. Adapted Diabetes Complications Severity Index (aDCSI) scores were computed for 1,061,140 unique adult patients with a diagnosis of diabetes. Severe complications were defined as those with an aDCSI ≥ 4. Population average models, estimated using generalized estimating equations, were used to identify individual- and area-level predictors of severe diabetes complications.

**Results:**

Non-Hispanic Black patients had the highest odds of severe diabetes complications compared to non-Hispanic White patients among both males (Odds Ratio [OR] = 1.20, 95% Confidence Interval [CI]: 1.17, 1.23) and females (OR = 1.27, 95% CI: 1.23, 1.31). Comorbidities associated with higher odds of severe complications included hypertension (OR = 2.30, 95% CI: 2.23, 2.37), hyperlipidemia (OR = 1.29, 95% CI: 1.27, 1.31), obesity (OR = 1.24, 95% CI: 1.21, 1.26) and depression (OR = 1.09, 95% CI: 1.07, 1.11), while the odds were lower for patients with a diagnosis of arthritis (OR = 0.81, 95% CI: 0.79, 0.82). Type of health insurance coverage was associated with the severity of diabetes complications, with significantly higher odds of severe complications among Medicare (OR = 1.85, 95% CI: 1.80, 1.90) and Medicaid (OR = 1.83, 95% CI: 1.77, 1.90) patients compared to those with private insurance. Residing within the least socioeconomically deprived ZIP code tabulation areas (ZCTAs) in the state had a protective effect compared to residing outside of these areas.

**Conclusions:**

Racial, ethnic, and socioeconomic disparities in the severity of diabetes complications exist among hospitalized patients in Florida. The observed disparities likely reflect challenges to maintaining glycemic control and managing cardiovascular risk factors, particularly for patients with multiple chronic conditions. Interventions to improve diabetes management should focus on populations with disproportionately high burdens of severe complications to improve quality of life and decrease premature mortality among adult patients with diabetes in Florida.

**Supplementary Information:**

The online version contains supplementary material available at 10.1186/s12889-023-17288-x.

## Background

Diabetes mellitus is one of the most common chronic diseases in the United States (U.S.), with an estimated 28.5 million diagnosed cases among adults in 2019 [1]. Complications of diabetes are a significant public health burden [2], and result in substantial economic costs to patients and healthcare systems [3, 4]. Short-term effects of uncontrolled diabetes include acute metabolic complications such as diabetic ketoacidosis or hypoglycemic coma [5]. Long-term effects of diabetes, on the other hand, are associated with both macrovascular and microvascular complications. The macrovascular complications include cardiovascular disease, peripheral vascular disease, and stroke [5, 6], while microvascular complications include retinopathy, neuropathy and nephropathy [5]. Retinal lesions may lead to progressive visual impairment resulting in blindness, while neuropathy increases the risk of lower-extremity ulcerations that may necessitate amputation [5, 6]. Nephropathy can ultimately progress to end-stage renal disease, requiring chronic dialysis treatment or kidney transplantation [5, 6]. Such complications can permanently impact quality of life [7–9], and are associated with depression [10], functional limitations, and premature mortality [6, 11, 12]. Therefore, the primary goal of diabetes care is to prevent complications through glycemic control and management of cardiovascular risk factors [13–15].

Hospitalizations due to complications of diabetes, which is considered an ambulatory care sensitive condition, often reflect problems with access to or utilization of quality outpatient care [16–19]. Therefore, reducing the burden of potentially avoidable diabetes-related complications represents an important opportunity to improve population health in the United States. This is particularly true in southeastern states, which have the highest burden of diabetes in the nation [20, 21]. Florida is the most populous state in this region, and it is estimated that at least 2.5 million of the state’s adult residents have diabetes [22, 23]. While hospitalizations due to diabetes complications may be prevented with timely access to care and effective management [16], there have been substantial increases in diabetes-related hospitalizations, amputations, and hospital costs in Florida in recent years [24]. Complications of diabetes will likely present an ongoing challenge in Florida due to the state’s growing population of adults aged 65 and over [25]. Age is a known risk factor for Type 2 diabetes, which represents 90–95% of the diabetes burden in the U.S. [6]. Older adults account for the majority of healthcare resource utilization attributable to diabetes [4], and are also projected to account for most of the future increase in diabetes prevalence in the U.S. [26]. Reducing the burden of preventable diabetes-related complications represents an important opportunity to meaningfully improve the health of Floridians.

Evidence of disparities in diabetes prevalence and participation in diabetes preventive programs (diabetes self-management education) has been reported in Florida [27, 28], suggesting that there might also be disparities in complications of diabetes in the state. However, there is a lack of information on distribution or determinants/predictors of diabetes complications in Florida. Since severe complications of diabetes pose the greatest burden to patients and health systems [29], identifying determinants/predictors of these complications is important for guiding strategies aimed at reducing the burden. Therefore, the objective of this study was to identify individual- and area-level predictors of severe diabetes complications among hospitalized adult patients in Florida.

## Methodology

### Ethics approval and consent to participate

This study was performed in accordance with the Declaration of Helsinki and was approved by the University of Tennessee Institutional Review Board (Number: UTK IRB-22-07182-XP). In accordance with 45 CFR 46.116(f), informed consent was waived by the University of Tennessee Institutional Review Board. In addition, the request for waiver of HIPAA authorization for the conduct of the study was approved the University of Tennessee Institutional Review Board.

### Study area

This study was conducted in the state of Florida, which is located in the Southeastern United States and comprises 67 counties and 983 ZIP code tabulation areas (ZCTAs) [30]. In 2019, Florida had an estimated population of 20.9 million people, with 20.1% of the population aged 65 and older [31]. ZCTA-level population ranged from 0 to 76,508 residents [31]. An estimated 14.0% of Floridians had income below the poverty level in 2019, and 19.5% of adults under the age of 65 did not have health insurance coverage [32, 33]. In 2019, the estimated statewide prevalence of diabetes among adults in Florida was 11.7% [34].

### Data sources and preparation

#### Patient-level variables

Hospital discharge data from 2016 to 2019 were obtained from the Florida Agency for Health Care Administration through a Data Use Agreement with the Florida Department of Health. Data management was performed using SAS 9.4 [35]. Variables extracted included masked Social Security Number (SSN), patient age, sex, race and ethnicity, admission and discharge dates, principal payer, patient ZIP code and county of residence, principal International Classification of Diseases, Tenth Revision, Clinical Modification (ICD-10-CM) diagnosis code, and all other ICD-10-CM diagnosis codes. Records of hospitalized patients between the ages of 18 and 100 with a diagnosis of Type 1 or Type 2 diabetes mellitus (ICD-10-CM codes E10.x or E11.x) in any diagnosis field were selected for inclusion in the study, yielding 2,581,031 hospitalizations. Dichotomous variables were generated for each entry to indicate the presence/absence of a diagnosis for each of the following co-morbidities: hypertension, hyperlipidemia, obesity, depression, and arthritis. Diagnosis codes used to construct each of the comorbidity variables are displayed in Table 1.

**Table 1:** Diagnosis codes used to identify selected comorbidities among hospitalized patients with diabetes

Condition	ICD-10-CM Diagnosis Codes
Hypertension	I10.x, I11.x, I12.x, I13.x, I15.x
Hyperlipidemia	E78.00, E78.01, E78.1, E78.2, E78.3, E78.41, E78.49, E78.5
Obesity	Z68.54, Z68.3, Z68.4, Z68.35, Z68.36, Z68.37, Z68.38, Z68.39, E66.01, E66.2, E66.09, E66.1, E66.8, E66.9
Depression	F31.3-6, F32.x, F33.0-3, F33.40-42, F33.8, F33.9, F34.1
Arthritis	M05.x, M06.x, M12.0x, M15.x, M16.x, M17.x, M18.x, M19.x

Principal payer was classified into the following groups: Medicare (patients with Medicare or Medicare managed care plans); Medicaid (Medicaid, Medicaid managed care, or Kidcare); private insurance (commercial health insurance, worker’s compensation, or commercial liability coverage); Veterans Affairs (VA), TriCare, federal, state and local government; self-pay (patients with no health insurance coverage); non-payment, and other. The non-payment designation applies to several categories, including clinical trials or other research, charity, and refusal to pay or debt.

Adapted Diabetes Complications Severity Index (aDCSI) scores were calculated for each hospitalization using the ICD-10-CM diagnosis codes [29, 36, 37]. The DCSI is a validated 14-level (scores of 0 to 13) measure that is a better predictor of mortality than a simple count of number of diabetic complications a patient has [29, 36]. Seven categories of diabetes complications are included in the computation of DCSI score: cardiovascular, cerebrovascular, metabolic, renal, neurologic, ophthalmic, and peripheral vascular [36]. Complications for each of the seven categories are assigned scores from 0 to 2 based on presence and severity (with the exception of neuropathy, which has a maximum of 1). These are generated from ICD-10-CM codes and renal laboratory parameters, and are summed to yield the patient’s total DCSI score [29, 36]. The adapted DCSI (aDCSI) does not include laboratory parameters, but is otherwise identical to the original DCSI and exhibits similar performance [37]. In order to identify patients with severe complications, aDCSI score was categorized into a dichotomous variable (aDCSI < 4 and aDCSI ≥ 4). An aDCSI score ≥ 4 indicates that a patient: (a) has at least one complication related to the majority of the body systems assessed in the index, and/or (b) has one or more complications classified as severe.

A single admission was randomly selected for each patient with a unique identifier (masked SSN) to limit potential bias from patients with repeated hospitalizations during the study period. A total of 83,843 entries did not include a unique patient identifier, indicating that the patient did not provide an SSN upon admission to the hospital. Findings of initial descriptive analyses suggested these patients differed from those who did report SSNs with respect to several characteristics, and therefore these entries were retained as their exclusion could bias the findings of the analysis. Furthermore, recent research suggests that hospital patients who do not report an SSN tend to be from vulnerable sub-populations that may be at higher risk of adverse health outcomes compared to the general population [38].

Since individual identifiers were not present to enable selection of unique patients from this group of entries, a matching scheme was employed to link records that were most likely to have originated from a single patient (Appendix 1). Records were matched based on patient age, gender, diabetes type, ZIP code, race, and ethnicity (where race and ethnicity matched between records but could be unknown). In total, 43,807 unique patients were identified from this group, and a single entry was randomly selected from each for further analysis.

#### ZCTA-level variables

Patient ZIP codes were joined to ZIP code tabulation areas (ZCTAs) using the Uniform Data System (UDS) Mapper ZIP code to ZCTA crosswalk [39]. Fifteen ZCTAs with total populations of fewer than 100 people were excluded to minimize bias from areas with very low population counts [40], resulting in the exclusion of 1.5% of ZCTAs and 0.045% of patients (*n* = 464) from the study. Theoretical domains and data sources for ZCTA-level variables investigated are listed in Table 2. Each ZCTA was classified as either rural or non-rural based on locale assignments published by the National Center for Education Statistics [41].

Physician information extracted from the Florida Department of Health, Division of Medical Quality Assurance’s Florida Healthcare Practitioner Data Portal [42] included physician ID, specialty, practice ZIP code, county and state, license status, activity description, and dates of issuance and expiration. Physicians with internal medicine or family practice specialties were considered primary care physicians. Pharmacy data were extracted from the same portal, and included practice address ZIP code and state, dates of license issuance and expiration, and pharmacy status. Additional information about the physician and pharmacy data used in this study may be found in Appendix 1. Physician practice address and pharmacy ZIP codes were joined to ZCTAs, and the number of primary care physicians and pharmacies were aggregated to the ZCTA level. Total population, obtained from the U.S. Census Bureau American Community Survey 5-year estimates for 2015–2019 [43], was used to compute the number of practicing primary care physicians and pharmacies per 10,000 population in each ZCTA.

The number of grocery stores or supermarkets, convenience stores, and limited service (fast food) restaurants at the ZIP code level were obtained from the U.S. Census Bureau 2016 Annual Economic Survey [44, 45]. Food establishment data were joined to ZCTAs [39]. Land area of each ZCTA was extracted from U.S. Census Bureau TIGER/Line shapefiles, and used to calculate pharmacy, supermarket, convenience store, and fast food restaurant density [46].

**Table 2:** Theoretical domains and data sources of ZCTA-level variables investigated as predictors of severe diabetes complications

Variable names and domains	Data sources
***Rurality***	
ZCTA^a^ locale designations	U.S. Department of Education National Center for Education Statistics – Education Demographic and Geographic Estimates (EDGE) Program
***Healthcare access***	
Number of primary care physicians per 10,000 population	Florida Healthcare Practitioner Data Portal, American Community Survey (ACS) 2015–2019 5-year estimates (Table DP05)
Number of pharmacies per 10,000 population	
***Food environment***	
Number of grocery stores or supermarkets per km^2^	U.S. Census Bureau 2016 Annual Economic Survey (Table CB1600ZBP), U.S. Census Bureau TIGER/Line Shapefiles
Number of convenience stores per km^2^	
Number of limited service (fast food) restaurants per km^2^	
***Education***	
Percentage of population 25 years or older with less than a high school education	ACS 2015–2019 5-year estimates (Table S1501)
***Employment***	
Unemployment rate in population 16 years and older	ACS 2015–2019 5-year estimates (Table S2301)
Percentage of males aged 20–64 not participating in the labor force	
***Housing***	
Percentage renter-occupied housing units	ACS 2015–2019 5-year estimates (Table DP04)
Percentage vacant housing units	ACS 2015–2019 5-year estimates (Table DP04)
Percentage occupied housing units with > 1 occupant per room	ACS 2015–2019 5-year estimates (Table S2501)
Percentage renter- and owner-occupied households where gross rent or selected monthly owner costs are at least 50% of household income	ACS 2015–2019 5-year estimates (Table B25070 and B25095)
Median value of owner-occupied housing units	ACS 2015–2019 5-year estimates (Table DP04)
***Occupation***	
Percentage of employed males 16 and older with management occupations	ACS 2015–2019 5-year estimates (Table S2401)
Percentage of employed males 16 and over with professional occupations	
Percentage of employed females 16 and over with management occupations	
Percentage of employed females 16 and over with professional occupations	
***Poverty***	
Percent of families with income below poverty level	ACS 2015–2019 5-year estimates (Table S1702)
Percentage of female-headed households with own children under 18 years of age	ACS 2015–2019 5-year estimates (Table S1101)
Percentage of households with annual income of less than $30,000	ACS 2015–2019 5-year estimates (Table B19001)
Percentage of families with public assistance income or in households receiving Supplemental Nutrition Assistance Program (SNAP) benefits	ACS 2015–2019 5-year estimates (Table B19123)
Percentage of occupied households with no vehicles available	ACS 2015–2019 5-year estimates (Table DP04)
***Racial composition***	
Percentage non-Hispanic Black residents	ACS 2015–2019 5-year estimates (Table DP05)
***Residential stability***	
Percentage of current residents living in the same house as 1 year ago	ACS 2015–2019 5-year estimates (Table B07001)
Percentage of residents 65 years and older	ACS 2015–2019 5-year estimates (Table DP05)

^a^ZIP code tabulation area

### Neighborhood deprivation index

Neighborhood Deprivation Index (NDI) values were computed using the method developed by Messer and colleagues [47]. Numerous studies have used the NDI in analyses investigating associations between area-level socioeconomic status and health-related behaviors or outcomes at both the census tract [48–52], and ZCTA levels [53–55].

Twenty census variables representing the following domains were assessed for inclusion in the NDI: education, employment, housing, occupation, poverty, racial composition, and residential stability [47]. Individual variables and their data sources are listed in Table 2. Forty-one (4.24%) of the 968 ZCTAs included in the study did not have complete data for all twenty NDI variables. Seventeen continuous variables had one or more missing values, with a maximum of 38 missing observations (for the median household value variable).

Due to the missing data, multiple imputation (*m* = 5) with fully conditional specification was performed using the “mice” package in R [56]. Since many of the variables had significant skewness, imputation with predictive mean matching was used [57, 58]. The imputation model for each variable with missing data included the remaining nineteen NDI variables, as well as the rurality variable.

Computation of the NDI involved principal components analysis (PCA) and selection of a subset of candidate census variables that contributed highly to the first component, which was then retained for further analysis [47]. The NDI scores were standardized to have a mean of 0 and standard deviation of 1, and categorical variables representing NDI tertiles, quartiles, and quintiles were generated.

### Statistical analysis

To investigate and identify predictors of severe diabetes complications (aDCSI ≥ 4), population average generalized linear models were fit to the data using generalized estimating equations (GEE), specifying binomial distribution, logit link, and an exchangeable working correlation structure [59, 60]. Patients residing within the same ZCTA were treated as clustered observations. Modeling was performed in SAS 9.4 using the GENMOD procedure [35]. First, univariable models were fit to identify potential predictors, and variables associated with the outcome at a liberal *p-*value of < 0.15 were considered for inclusion in multivariable models. Correlations between potential predictor variables were assessed using Spearman rank-order correlation. If a pair of variables were highly correlated (|*r*_*s*_| > 0.7), then only one was selected for inclusion in the modeling process.

After identification of candidate predictor variables, models with patient-level predictors were fit using backward selection and an inclusion criterion of *p* < 0.05. Changes in the modified quasi-likelihood information criterion (QIC_u_), were also used to guide variable selection, with lower values indicating a model with better fit to the data [59]. If removal of a variable from the model changed the magnitude of another variable’s coefficient by more than 20%, it was deemed a potential confounder and considered for retention in the model regardless of its statistical significance. Patient-level variables considered as potential predictors included year of admission, age, gender, race/ethnicity, health insurance coverage, diabetes type, and comorbidities (hypertension, arthritis, hyperlipidemia, depression, and obesity). Since patients who do not report an SSN during hospitalization tend to be from socioeconomically vulnerable groups with low access to healthcare services [38], the presence/absence of an SSN was also assessed as a potential predictor. Age was assessed as both a continuous variable, scaled by subtracting 18 (the minimum age for inclusion), and as a categorical variable. A quadratic term for the continuous age variable was also assessed for significance. An interaction term for gender and race/ethnicity was assessed, based upon findings of previous research [61].

After specification of the patient-level variables in the model, significant ZCTA-level predictors were identified using forward selection with a cutoff of *p* < 0.05, guided by changes in QIC_u_. Due to high correlations between food environment and healthcare access variables, physicians per capita and grocery store density were the only predictors from these categories considered for inclusion in the model. Rurality, as well as NDI tertiles, quartiles, and quintiles were also assessed. In addition, since racial composition was not incorporated into the NDI variable, which was only moderately correlated with percentage non-Hispanic Black residents, this was also considered as a potential predictor.

Results for each step in the model-building process for the five imputed datasets were combined using the MIANALYZE procedure in SAS [35]. Results of partial F-tests assessing the overall significance of categorical variables with more than two levels were combined using the “miceadds” package in R [62].

## Results

### Descriptive statistics

A total of 1,061,140 hospital records for unique patients between the ages of 18 and 100 with diagnoses of Type 1 or Type 2 diabetes mellitus were included in the study. The median duration of hospitalization was 3 days, with an interquartile range (IQR) of 2 to 6 days and an overall range of 0 to 1,143 days. Median age was 69 years (IQR 58 to 78), and there were more male patients (51.7%) than female patients (48.3%). Most patients were non-Hispanic White (60.6%), followed by Hispanic patients (18.7%) and non-Hispanic Black or African American patients (17.7%) (Table 3). The vast majority (97.3%) of patients had Type 2 diabetes, and the most common comorbidities were hypertension (83.1%) and hyperlipidemia (55.6%). Medicare (64.9%), private insurance (18.3%), and Medicaid (7.5%) were the most frequently listed payment sources. Adapted DCSI (aDCSI) scores ranged from 0 to 11, with a median of 1 and IQR of 0 to 2. Approximately one-tenth (9.8%) of patients in the study had an aDCSI score of 4 or higher.

**Table 3:** Characteristics and Adapted Diabetes Complications Severity Index (aDCSI) scores of hospitalized adult patients with diabetes

Characteristic	% (n)	aDCSI < 4	aDCSI≥4
Year of admission			
2016	24.4 (258,916)	24.3 (232,982)	25.0 (25,934)
2017	23.9 (253,149)	24.0 (229,394)	22.9 (23,755)
2018	24.8 (263,102)	24.9 (237,982)	24.2 (25,120)
2019	27.0 (285,973)	26.8 (256,833)	28.0 (29,140)
Gender			
Male	51.7 (548,773)	50.8 (486,685)	59.7 (62,088)
Female	48.3 (512,367)	49.2 (470,506)	40.3 (41,861)
Race/ethnicity			
Non-Hispanic American Indian/ Alaska Native	0.13 (1,348)	0.13 (1,231)	0.11 (117)
Non-Hispanic Asian	0.98 (10,128)	1.00 (9,343)	0.77 (785)
Non-Hispanic Black	17.7 (183,600)	17.6 (164,514)	18.8 (19,086)
Non-Hispanic Hawaiian/ Pacific Islander	0.05 (479)	0.05 (436)	0.04 (43)
Non-Hispanic White	60.6 (628,604)	60.1 (562,971)	64.5 (65,633)
Other non-Hispanic	1.9 (20,123)	1.96 (18,350)	1.74 (1,773)
Hispanic	18.7 (193,754)	19.2 (179,394)	14.1 (14,360)
Presence of social security number (SSN)			
SSN provided on admission	95.9 (1,017,333)	95.8 (916,990)	96.5 (100,343)
No SSN on admission	4.1 (43,807)	4.2 (40,201)	3.5 (3,606)
Principal payer			
Medicare	64.9 (689,067)	63.3 (606,108)	79.8 (82,959)
Medicaid	7.5 (79,339)	7.7 (73,229)	5.9 (6,110)
Private	18.3 (193,909)	19.3 (184,498)	9.1 (9,411)
VA^a^, TriCare, federal, state or local government	2.7 (28,929)	2.8 (26,523)	2.3 (2,406)
Self-pay (uninsured)	4.7 (49,808)	5.0 (47,792)	1.9 (2,016)
Non-payment	1.5 (16,073)	1.6 (15,406)	0.64 (667)
Other	0.4 (4,015)	0.38 (3,635)	0.37 (380)
Diabetes type			
Type 1	2.7 (28,815)	2.7 (25,557)	3.1 (3,258)
Type 2	97.3 (1,032,325)	97.3 (931,634)	96.9 (100,691)
Diagnosed comorbidities			
Hypertension	83.1 (882,253)	82.0 (784,959)	93.6 (97,294)
Arthritis	14.5 (153,460)	14.5 (139,133)	13.8 (14,327)
Hyperlipidemia	55.6 (590,247)	54.5 (521,237)	66.4 (69,010)
Obesity	26.8 (284,369)	26.7 (255,464)	27.8 (28,905)
Depression	12.6 (133,262)	12.5 (119,797)	13.0 (13,465)
Neighborhood Deprivation Index quartiles			
1 (most deprived)	26.4 (280,228)	26.5 (253,793)	25.4 (26,436)
2	29.9 (317,117)	29.8 (285,679)	30.2 (31,438)
3	25.8 (273,824)	25.7 (246,411)	26.4 (27,413)
4 (least deprived)	17.9 (189,971)	17.9 (171,309)	18.0 (18,662)

^a^Veterans Affairs

The number of patients from each ZCTA ranged from 1 to 5,162 with a median of 907 and IQR of 402 to 1,625. Summary statistics of ZCTA-level characteristics, all of which were non-normally distributed, are displayed in Table 4. In 2016, the median numbers of primary care physicians and licensed pharmacies were 6 (IQR 1.7 to 13.6) and 3.1 (IQR 1.3 to 5.5) per 10,000 population, respectively. The percentage of residents with educational attainment below the high school level ranged from 0 to 62.5%, with a median of 10.3%. Other socioeconomic characteristics of ZCTAs also varied widely; for instance, the median unemployment rate was 5.1%, but ranged from 0 to 78.8%. Median value of owner-occupied housing units ranged from $12,800 to $1,208,3300 (median $188,740, IQR $130,420 to $273,460). There was a higher median percentage of females in professional occupations (37.9%) compared to males (28.8%), but the percentage of males in management positions (median 10.6%) tended to be higher than females (median 8.0%). The median percentage of households in poverty was 8.9%, but ranged from 0 to 70.8%. The percentage of non-Hispanic Black residents at the ZCTA level also ranged widely, from 0 to 100%, with a median of 7.9%. Residential stability within a one-year period tended to be high (median 85.6%, IQR 81.8 to 88.6%).

**Table 4:** Characteristics of ZIP code tabulation areas in Florida, USA

Variable Name and Year	Quartiles				
	Min.	Q1	Median	Q3	Max.
Total population (2015–2019)	104	7,940.5	19,030.5	31,342.5	76,508
***Healthcare Access (2016)***					
Primary care physicians / 10,000 pop.	0	1.7	6.0	13.6	7,541
Pharmacies / 10,000 pop.	0	1.3	3.1	5.5	390.2
***Food Environment (2016)***					
Grocery stores / km^2^	0	0.003	0.05	0.2	7.6
Convenience stores / km^2^	0	0	0.02	0.1	6.7
Limited service restaurants / km^2^	0	0.01	0.2	0.8	38.2
***Education (2015–2019)***					
% ≥ 25 with less than high school education	0	6.3	10.3	16.7	62.5
***Employment (2015–2019)***					
Unemployment rate in population 16 years and older	0	3.8	5.1	7.0	78.8
% males no longer in work force	0	14.4	19.0	26.1	100
***Housing (2015–2019)***					
% renter-occupied housing units	0	19.1	27.5	41.2	100
% vacant housing units	0	10.5	16.1	24.0	97.6
% crowded (> 1 person/room)	0	0.9	2.0	3.7	47.2
% renter/owner costs > 50% of income	0	10.6	13.9	18.2	49.8
Median value, owner-occupied housing units	$12,800	$130,420	$188,740	$273,460	$1,208,300
***Occupation (2015–2019)***					
% males in management	0	6.9	10.6	14.6	100
% males in professional occupations	0	20.4	28.8	39.4	100
% females in management	0	5.4	8.0	10.8	100
% females in professional occupations	0	31.6	37.9	45.8	100
***Poverty (2015–2019)***					
% households in poverty	0	5.3	8.9	13.8	70.8
% female-headed households with dependent children	0	3.1	5.1	7.5	66.0
% households earning < $30,000 / year	0	18.5	25.7	33.4	100
% households receiving public assistance or SNAP^a^ benefits	0	6.7	13.1	21.2	100
% households with no car	0	2.5	4.5	7.5	41.2
***Racial composition (2015–2019)***					
% non-Hispanic Black residents	0	2.8	7.9	17.2	100
***Residential stability (2015–2019)***					
% in same residence as 1 year ago	11.9	81.8	85.6	88.6	100
% 65 years and over	0	13.8	19.2	27.1	87.1

^a^Supplemental Nutrition Assistance Program

### Neighborhood deprivation index

The Neighborhood Deprivation Index (NDI) included variables from the following domains: education (percentage of residents at least 25 years of age with education below the high school level), housing (median value of owner-occupied housing units), occupation (percentage of employed males and females 16 and over with management or professional occupations), poverty (percentage of families with income below the poverty level, percentage of households with annual income less than $30,000, percentage of families with public assistance income or in households receiving Supplemental Nutrition Assistance Program (SNAP) benefits), and residential stability (percentage of residents aged 65 and over). Variable loadings on the first principal component, which were used to construct NDI scores, are displayed in Table 5. Across the entire study area, 59.6% of the variance of the ten variables in the index was explained by the first principal component. When PCA was stratified by rurality, the percentage of variance explained was higher for non-rural ZCTAs (65.7%) than for rural ZCTAs (53.6%). Characteristics of the ZCTAs in each NDI quartile are summarized in Table 6. Lower values of the index were associated with higher percentages of the population with less than a high school education, families with income below the poverty level, households with annual income less than $30,000, and families receiving public assistance income. Higher median housing values, percentage of employed males and females with professional or management occupations, and percentage of older adults in the population were associated with higher values of the NDI. Therefore, lower values of the index indicate more deprived areas. Fewer patients (17.9%) resided in ZCTAs in the highest (least deprived) NDI quartile compared to all other quartiles (Table 3).

**Table 5:** Results of principal components analysis used to construct Neighborhood Deprivation Index for Florida ZCTAs

Theoretical domains and variable names	Variable loadings on first principal component		
	Rural ZCTAs^a^	Non-rural ZCTAs^a^	All ZCTAs^a^
***Education***			
% ≥ 25 with less than high school education	-0.78	-0.91	-0.84
***Housing***			
Median value, owner-occupied housing units	0.68	0.58	0.63
***Occupation***			
% males in management	0.57	0.73	0.63
% males in professional occupations	0.76	0.77	0.77
% females in management	0.36	0.62	0.48
% females in professional occupations	0.60	0.70	0.67
***Poverty***			
% of families with income below poverty level	-0.77	-0.90	-0.83
% households with annual income < $30,000	-0.83	-0.89	-0.86
% families with public assistance income or in households receiving SNAP^b^ benefits	-0.82	-0.98	-0.89
***Residential stability***			
% of residents 65 years and older	0.22	0.27	0.24
**Percent of variance explained**	53.6%	65.7%	59.6%

^a^ZIP code tabulation areas

^b^Supplemental Nutrition Assistance Program

**Table 6:** Median values of ZIP code tabulation area characteristics by Neighborhood Deprivation Index (NDI) quartile

Variable	NDI quartile			
	1 *(most deprived)*	2	3	4 *(least deprived)*
% ≥ 25 with less than high school education	20.6	12.7	8.5	4.4
Median value, owner-occupied housing units	$105,580	$157,250	$207,160	$347,240
% males in management	5.6	8.9	11.8	17.9
% males in professional occupations	16.5	25.8	32.5	47.4
% females in management	5.1	6.7	9.0	12.0
% females in professional occupations	28.7	35.0	41.2	50.5
% families with income below poverty level	17.8	10.8	7.1	4.2
% households with annual income < $30,000	38.4	29.1	22.2	16.0
% families with public assistance income or in households receiving SNAP^a^ benefits	27.3	16.8	10.3	4.0
% residents 65 years and older	16.5	19.6	19.7	21.8

^a^Supplemental Nutrition Assistance Program

### Population average generalized linear model

Significant individual-level predictors of severe diabetes complications (aDCSI score ≥ 4) included age, gender, race/ethnicity, diabetes type, comorbidities (hypertension, hyperlipidemia, obesity, depression and arthritis), payer, report of an SSN upon admission, and year of admission (Tables 7 and 8).

**Table 7:** Predictors of severe diabetes complications among hospitalized adults in Florida, USA, 2016–2019

Parameters	Coefficient	95% Confidence Interval*		*p*-value
Intercept	-4.416	-4.496	-4.335	< 0.0001
Age	0.040	0.037	0.043	< 0.0001
Age (quadratic)	-0.0002	-0.0002	-0.0002	< 0.0001
Gender				
Male	0.398	0.381	0.416	< 0.0001
Female	Ref.	-	-	-
Race/ethnicity				< 0.0001
Non-Hispanic American Indian/Alaska Native	0.123	-0.151	0.397	0.3777
Non-Hispanic Asian	-0.088	-0.183	0.008	0.0718
Non-Hispanic Black or African American	0.238	0.207	0.268	< 0.0001
Non-Hispanic Hawaiian/ Pacific Islander	0.098	-0.383	0.578	0.6906
Other non-Hispanic	-0.070	-0.149	0.009	0.0812
Hispanic	-0.243	-0.277	-0.210	< 0.0001
Non-Hispanic White	Ref.	-	-	-
Diabetes type				
Type 2	-0.976	-1.017	-0.934	< 0.0001
Type 1	Ref.	-	-	-
Comorbidities				
Hypertension	0.833	0.804	0.862	< 0.0001
Hyperlipidemia	0.258	0.243	0.273	< 0.0001
Obesity	0.211	0.194	0.229	< 0.0001
Depression	0.089	0.069	0.109	< 0.0001
Arthritis	-0.216	-0.237	-0.194	< 0.0001
Principal payer				< 0.0001
Medicare	0.615	0.587	0.642	< 0.0001
Medicaid	0.606	0.568	0.644	< 0.0001
VA^a^, TriCare, gov’t	0.285	0.233	0.337	< 0.0001
Uninsured	-0.043	-0.096	0.010	0.1104
Non-payment	-0.019	-0.106	0.067	0.6586
Other	0.450	0.337	0.562	< 0.0001
Private	Ref.	-	-	-
Presence of social security number (SSN)				
No SSN on admission	0.105	0.067	0.143	< 0.0001
SSN on admission	Ref.	-	-	-
Admission year				< 0.0001
2017	-0.086	-0.105	-0.066	< 0.0001
2018	-0.067	-0.088	-0.049	< 0.0001
2019	-0.011	-0.030	0.008	0.2613
2016	Ref.	-	-	-
Neighborhood Deprivation Index quartile				
Lower 3 quartiles	0.090	0.064	0.117	< 0.0001
Least deprived quartile	Ref.	-	-	-
*Interaction term*				< 0.0001
Male				
Non-Hispanic American Indian/ Alaska Native	-0.233	-0.615	0.148	0.2299
Non-Hispanic Asian	-0.147	-0.289	-0.005	0.0421
Non-Hispanic Black or African American	-0.055	-0.092	-0.019	0.0033
Non-Hispanic Hawaiian/ Pacific Islander	-0.198	-0.860	0.464	0.5579
Other non-Hispanic	0.091	-0.011	0.194	0.0817
Hispanic	0.098	0.059	0.136	< 0.0001
Female, non-Hispanic White	Ref.	-	-	-

*Confidence intervals computed using modified sandwich estimate of variance

^a^Veterans Affairs

**Table 8:** Odds ratios for predictors of severe diabetes complications among hospitalized adults in Florida, USA, 2016–2019

Parameters	Odds Ratio	95% Confidence Interval*		*p*-value
Intercept	0.012	0.011	0.013	< 0.0001
Age	1.041	1.038	1.044	< 0.0001
Age (quadratic)	0.9998	0.9998	0.9998	< 0.0001
Gender & race/ethnicity				
* Males*				
Non-Hispanic American Indian/Alaska Native	0.896	0.666	1.205	0.4675
Non-Hispanic Asian	0.791	0.716	0.873	< 0.0001
Non-Hispanic Black or African American	1.200	1.168	1.233	< 0.0001
Non-Hispanic Hawaiian/ Pacific Islander	0.904	0.587	1.395	0.3326
Other non-Hispanic	1.021	0.958	1.089	0.5223
Hispanic	0.864	0.840	0.889	< 0.0001
Non-Hispanic White	Ref.	-	-	-
* Females*				
Non-Hispanic American Indian/Alaska Native	1.131	0.860	1.487	0.3777
Non-Hispanic Asian	0.916	0.833	1.008	0.0718
Non-Hispanic Black or African American	1.269	1.230	1.307	< 0.0001
Non-Hispanic Hawaiian/ Pacific Islander	1.103	0.682	1.782	0.6906
Other non-Hispanic	0.932	0.862	1.009	0.0812
Hispanic	0.784	0.758	0.811	< 0.0001
Non-Hispanic White	Ref.	-	-	-
Diabetes type				
Type 2	0.377	0.362	0.393	< 0.0001
Type 1	Ref.	-	-	-
Comorbidities				
Hypertension	2.300	2.234	2.368	< 0.0001
Hyperlipidemia	1.294	1.275	1.314	< 0.0001
Obesity	1.235	1.214	1.257	< 0.0001
Depression	1.093	1.071	1.115	< 0.0001
Arthritis	0.806	0.789	0.824	< 0.0001
Principal payer				
Medicare	1.850	1.799	1.900	< 0.0001
Medicaid	1.833	1.765	1.904	< 0.0001
VA^a^, TriCare, gov’t	1.330	1.262	1.401	< 0.0001
Uninsured	0.958	0.908	1.010	0.1104
Non-payment	0.981	0.899	1.069	0.6586
Other	1.568	1.401	1.754	< 0.0001
Private	Ref.	-	-	-
Presence of social security number (SSN)				
No SSN on admission	1.111	1.069	1.154	< 0.0001
SSN on admission	Ref.	-	-	-
Admission year				
2017	0.918	0.900	0.936	< 0.0001
2018	0.935	0.916	0.952	< 0.0001
2019	0.989	0.970	1.008	0.2613
2016	Ref.	-	-	-
Neighborhood Deprivation Index quartile				
Lower 3 quartiles	1.094	1.066	1.124	< 0.0001
Least deprived quartile	Ref.	-	-	-

*Confidence intervals computed using modified sandwich estimate of variance

^a^Veterans Affairs

Coefficients for linear and quadratic age terms in the final model had opposite signs, such that the odds of severe diabetes complications were higher for older patients, but the magnitude of this effect diminished as age increased. Racial and ethnic differences in the odds of severe diabetes complications differed by gender. For instance, among males, the odds of severe complications were 21% lower for Asian patients compared to non-Hispanic White patients (95% confidence interval [CI]: 0.72, 0.87, *p* < 0.0001), but a significant difference was not observed among female patients (*p* = 0.0718). On the other hand, while Hispanic male patients also had lower odds of severe complications compared to non-Hispanic White male patients (odds ratio [OR] = 0.86, 95% CI: 0.84, 0.89, *p* < 0.0001), the magnitude of the difference between these groups was greater among females (OR = 0.78, 95% CI: 0.76, 0.81, *p* < 0.0001). In contrast, both male (OR = 1.20, 95% CI: 1.17, 1.23, *p* < 0.0001) and female (OR = 1.27, 95% CI: 1.23, 1.31, *p* < 0.0001) non-Hispanic Black patients had higher odds of severe diabetes complications compared to non-Hispanic White patients.

Several clinical factors were associated with the severity of diabetes complications among hospitalized adult patients with diabetes. For instance, the odds of severe diabetes complications were 62% lower for patients with Type 2 compared to Type 1 diabetes (95% CI: 0.36, 0.39, *p* < 0.0001). Diagnoses of hypertension (OR = 2.30, 95% CI: 2.23, 2.37, *p* < 0.0001), hyperlipidemia (OR = 1.29, 95% CI: 1.27, 1.31, *p* < 0.0001), obesity (OR = 1.24, 95% CI: 1.21, 1.26, *p* < 0.0001) and depression (OR = 1.09, 95% CI: 1.07, 1.11, *p* < 0.0001) were associated with higher odds of severe diabetes complications, while arthritis was associated with lower odds of severe complications (OR = 0.81, 95% CI: 0.79, 0.82, *p* < 0.0001).

Lack of health insurance coverage was not a significant predictor of the severity of diabetes complications when compared to private insurance (*p* = 0.1104). However, patients with Medicare (OR = 1.85, 95% CI: 1.80, 1.90, *p* < 0.0001), Medicaid (OR = 1.83, 95% CI: 1.77, 1.90, *p* < 0.0001), VA and other government insurance (OR = 1.33, 95% CI: 1.26, 1.40, *p* < 0.0001), and other insurance sources (OR = 1.57, 95% CI: 1.40, 1.75, *p* < 0.0001) did have higher odds of severe complications compared to private insurance. Absence of an SSN upon hospital admission was also positively associated with severe diabetes complications (OR = 1.11, 95% CI: 1.07, 1.15, *p* < 0.0001). Patients admitted in 2017 (OR = 0.92, 95% CI: 0.90, 0.94, *p* < 0.0001) and 2018 (OR = 0.94, 95% CI: 0.92, 0.95, *p* < 0.0001) had slightly lower odds of severe complications than those admitted in 2016.

Neighborhood Deprivation Index (NDI) quartile was the only significant ZCTA-level predictor in the final model. Compared to patients living in ZCTAs in the least deprived quartile, patients in all other NDI quartiles had significantly higher odds of severe diabetes complications (OR = 1.09, 95% CI: 1.07, 1.12, *p* < 0.0001). Food environment, healthcare access, and racial composition variables were not significantly associated with severity of diabetes complications.

## Discussion

This study investigated and identified predictors of severe complications among adult patients hospitalized with diabetes in Florida. The odds of severe complications increased with age, a finding that likely reflects duration of the condition, which is associated with macrovascular events in affected patients [63]. The observed association between Type 1 diabetes and severe complications is also likely related to duration of the condition, since Type 1 diabetes tends to have an earlier age of onset than Type 2 diabetes [5]. The diminishing magnitude of the strength of association between older age and severity of complications observed in this study might be due to survival bias since higher aDCSI values are associated with higher risks of death [29, 37].

Several comorbidities were positively associated with severe diabetes complications, highlighting the difficulties of managing multiple chronic conditions. Patients with multiple conditions face challenges with coordination and quality of care, financial costs, and adhering to complex self-management recommendations [64]. Hypertension and hyperlipidemia, two important cardiovascular risk factors, were predictors of severe diabetes complications, consistent with findings of a previous study which reported that hypertension and/or hypercholesterolemia in patients with diabetes were predictors of poor health status [65]. Interestingly, having a diagnosis of arthritis was associated with lower odds of severe complications. While most evidence suggests that multiple conditions are associated with poorer quality of care, this may vary depending upon the specific combination of illnesses [64]. Further investigation is warranted to understand the mechanism of the negative association between arthritis and severe diabetes complications observed in this study.

Higher odds of severe diabetes complications were observed among patients with a diagnosis of depression compared to those without this diagnosis. Previous research has identified a bi-directional association between diabetes and depression, and the two conditions share risk factors such as socioeconomic deprivation [66]. The association between depression and severe diabetes complications observed in the current study could reflect the impacts of diabetes complications on quality of life [66]. This finding may also have been driven by poorer adherence to treatment and self-management recommendations among patients with diabetes who also have depression [66]. The association observed in this study underscores the importance of depression screening for patients with diabetes, as well as ensuring linkage to additional care when indicated [66].

There were racial/ethnic and gender disparities in the odds of severe diabetes complications, with non-Hispanic Black patients having significantly higher odds of severe complications than non-Hispanic White patients. In addition to having higher odds of severe complications, non-Hispanic Black patients tended to be younger, with the lowest median age (62 years) of any group in the present study. In comparison, non-Hispanic White patients had a median age of 70 years. These findings are consistent with those of a New York City study that reported younger average age for Black patients with diabetes compared to White patients and lower relative proportions of Black patients in older age groups [40]. The observed disparities in age and odds of severe complications could, in part, reflect earlier onset of diabetes among Black patients in Florida. They may also indicate disparities in the provision and quality of diabetes care [67–69], and may suggest earlier diabetes-related mortality among Black patients [40].

While previous studies have reported lower levels of glycemic control [70] and a higher prevalence of complications [71, 72] among Hispanic patients with diabetes compared to White patients, Hispanic patients in this study had lower odds of severe complications, a difference that was most pronounced among females. Previous research investigating predictors of severe diabetes complications in a Medicare population had the opposite finding [61]. This discrepancy could, in part, reflect differences between the compositions of the Hispanic populations of Florida and the U.S. overall. Diabetes prevalence in the Hispanic population in the U.S. varies by country of origin, with the highest diabetes prevalence and diabetes-related mortality rates among those of Mexican descent [70, 73, 74], who represent a much smaller percentage of the Hispanic population in Florida (13.5%) compared to the U.S. overall (59.5%) [75]. Significantly lower diabetes prevalence and mortality have been reported among Cubans and Cuban Americans, the largest subgroup of the Hispanic population in Florida [75]. Given the evidence of inequalities in diabetes prevalence, control, and outcomes within the Hispanic population, considering this diverse population as a single group may mask important differences between subgroups [74, 76]. However, detailed ethnicity information was not recorded in the hospitalization data, precluding more detailed investigation in the current study.

There is mixed evidence regarding the occurrence of diabetes complications among Asian Americans, who experience higher incidence of some complications, such as end-stage renal disease, and lower incidence of others (lower extremity amputations) compared to their non-Hispanic White counterparts [72]. In this study, Asian male patients had lower odds of severe complications compared to non-Hispanic White males, but no such difference was observed for Asian female patients, despite findings of a previous study that reported lower diabetes prevalence among females in all Asian subgroups [77]. The observed finding may reflect gender-based differences in psychosocial factors, health behaviors, and access to healthcare as well as other resources necessary for effective management of the condition [67, 78, 79]. However, this finding could also be a result of relative sample size in the current study. In 2019, Asian Americans represented 2.6% of Florida’s population [31], and Asian patients comprised an even lower percentage of the study population (0.98%, 10,128 patients). It is also worth noting that previous research has reported differences in the prevalence of diabetes, as well as risk factors and comorbidities, between different subgroups of the Asian population [77, 80]. Therefore, it is possible that differences in the severity of diabetes complications existed between subgroups of Asian patients in the current study. Unfortunately, this could not be investigated in this study because more detailed patient information was not available.

The odds of severe complications were higher among both Medicaid and Medicare patients than those with private insurance. As of July 2022, Florida is one of twelve states that have not adopted the Medicaid eligibility expansion of the Affordable Care Act, and therefore non-elderly, low-income adults in the state must meet additional criteria to be eligible for Medicaid [81]. These criteria include blindness and disability [82], both of which can result from complications of diabetes, which may partially explain the observed association. In addition, given the income eligibility threshold for Medicaid recipients, these patients are likely to face additional financial barriers to securing the resources and care necessary for effective diabetes control, despite having health insurance coverage. The higher odds of severe diabetes complications among Medicare patients compared to those with private insurance may reflect the older age of Medicare-eligible patients, and longer duration of diabetes in these patients. In addition, end-stage renal disease, a potential consequence of diabetic nephropathy [5], is one of the eligibility criteria for Medicare [83], which may also partly explain the observed association.

Lack of health insurance was not significantly associated with having severe diabetes complications when compared to private health insurance coverage. This was somewhat surprising, since health insurance coverage is a predictor of receiving quality diabetes care [68, 84]. The reason for this finding is unclear, although it could reflect utilization of care. As inpatient hospitalization carries a large cost burden, those without health insurance coverage may decline necessary care for financial reasons. This may have, in part, accounted for the low level of uninsurance among patients in the study (4.7%) compared to the overall population of Florida, which has the fourth-highest percentage of uninsured individuals in the nation (13.4%), although this difference could also reflect the older age of the hospital patients in this study, most of whom were insured with Medicare. In addition, it is possible that some individuals who forego purchasing health insurance coverage due to cost may do so in part because of younger age and fewer health conditions, which could have contributed to the lack of observed association between uninsurance and severity of complications in this study. It is also important to note that only patients’ health insurance coverage at the time of hospitalization was available, and prior insurance status was unknown. Since diabetes complications may result from cumulative years of inadequate glycemic control and cardiovascular risk factor management [85], and previously uninsured patients may qualify for public health insurance due to end-stage renal disease, blindness, or disability, health insurance coverage throughout the life course may be a better predictor of this outcome. Regardless, the persistence of disparities in severe diabetes complications despite controlling for health insurance suggests that barriers to diabetes control outside of the healthcare setting should also be investigated. Indeed, socioeconomic disparities in avoidable hospitalizations and lower extremity amputations due to diabetes have been reported in Canada, a country with universal access to healthcare [86].

In addition to the observed association between Medicaid coverage and severe complications, two other findings of this study suggest that socioeconomic position is related to the occurrence of severe diabetes complications. First, the odds of severe complications were higher among patients not reporting a social security number (SSN) than among those that did. It is worth noting that patients who do not report an SSN tend to be from vulnerable groups who may have lower access to healthcare and other important resources than those who do. Such groups may include temporary workers, undocumented immigrants, and those experiencing homelessness [38]. The higher odds of severe diabetes complications in these patients in the current study suggests that they face barriers to effective diabetes management. In addition, neighborhood deprivation, which is a function of educational attainment, housing value, occupation, poverty, and residential stability, was also a predictor of severe diabetes complications. This association underscores the importance of the living environment, which can impact the ability of patients with diabetes to effectively manage the condition and prevent complications. Residents of more deprived neighborhoods may have fewer opportunities for education and employment, and face both social and physical barriers to engaging in recommended health behaviors [87].

### Strengths and limitations

This study investigated a large sample of hospitalized adult patients with diabetes in Florida who were diverse with respect to demographic characteristics, health insurance coverage, and socioeconomic status. Previous studies that have investigated claims data from a single insurance payer have limited generalizability to patients with different insurance sources or without health insurance coverage. The use of hospital data in this study also prevented bias associated with self-reporting, a limitation of survey data. In addition, while some studies investigating the association between diabetes-related outcomes and socioeconomic position have substituted area-level measures for individual income, this analysis accounted for the nested nature of the data using population average models estimated with generalized estimating Eqs. [88, 89].

However, this study is not without limitations. A limited number of individual-level variables that reflect individual socioeconomic position (health insurance coverage and presence of a Social Security Number) were available for analysis. Study findings may have also been affected by differences in healthcare utilization, since the financial burden of hospitalization may lead patients with limited resources to decline necessary care. Further investigation of emergency department visits and outpatient stays is warranted to assess the potential impact of healthcare utilization bias on the findings of this study. These limitations notwithstanding, this study provides valuable information for guiding the development and implementation of health programs in Florida to reduce disparities in preventable complications of diabetes in the state.

## Conclusions

This study identified racial, ethnic, gender and socioeconomic disparities in the severity of diabetes complications among hospitalized adult patients in Florida. The disproportionately high risk of severe complications in some groups may reflect earlier diabetes onset as well as challenges to achieving glycemic control and managing cardiovascular risk factors. In addition, study findings highlight the challenge of achieving sustained, effective management of diabetes for patients with multiple chronic conditions and the importance of depression as a predictor of adverse outcomes of diabetes. Since hospitalizations due to diabetes complications are preventable, the findings of this study are useful for guiding health programming aimed at improving health outcomes for patients with diabetes in Florida. Targeting populations with disproportionately high burdens of severe complications using evidence-based interventions to facilitate effective diabetes management in outpatient and community-based settings will help reduce complications, improve quality of life, and decrease premature mortality among patients with diabetes.

### Electronic supplementary material

Below is the link to the electronic supplementary material.

Supplementary Material 1

## Data Availability

The hospital discharge data used in this study belong to a third party (Florida Agency for Health Care Administration). The authors do not have the legal authority to share the data. Details of the data and how to request access are available from the Florida Agency for Health Care Administration (https://quality.healthfinder.fl.gov/Researchers/OrderData/order-data.aspx). The corresponding author, Dr. Agricola Odoi, can be contacted regarding request for study data.

## Abbreviations

aDCSI: Adapted Diabetes Complications Severity Index
DCSI: Diabetes Complications Severity Index
EDGE: Education Demographic and Geographic Estimates
GEE: Generalized Estimating Equations
ICD-10-CM: International Classification of Diseases, Tenth Revision, Clinical Modification
IQR: Interquartile Range
IRB: Institutional Review Board
NDI: Neighborhood Deprivation Index
PCA: Principal Components Analysis
QIC_u_: Modified Quasi-Likelihood Information Criterion
SAS: Statistical Analysis Software
SNAP: Supplemental Nutrition Assistance Program
SSN: Social Security Number
ACS: American Community Survey
TIGER: Topographically Integrated Geographic Encoding and Referencing
UDS: Uniform Data System US:United States
USA: United States of America
UTK: University of Tennessee, Knoxville
VA: Veterans Affairs
ZCTA: ZIP Code Tabulation Area
ZIP: Zone Improvement Plan
