# Supplementary material for: Investigation of predictors of severity of diabetes complications among hospitalized patients with diabetes in Florida, 2016–2019
Source: BMC Public Health. 2023 Dec 5;23:2424. doi: 10.1186/s12889-023-17288-x (PMC10698929; doi:10.1186/s12889-023-17288-x)
Supplement: Supplementary file 1 — Supplementary Material 1 [file 12889_2023_17288_MOESM1_ESM.docx]

**Appendix 1**

**Methods**

*Patient record matching scheme*

Three potential matching schemes were investigated: (1) age, gender, diabetes type, and ZIP code, (2) age, gender, diabetes type, ZIP code, race, and ethnicity (where neither race nor ethnicity were unknown), and (3) age, gender, diabetes type, ZIP code, race, and ethnicity (where race and ethnicity matched between records but could be unknown). Time between hospitalizations was taken into consideration when identifying an age match. To validate the matching schemes, each was applied to a test set of 2,370 entries from 1,000 randomly selected patients with known unique identifiers. Matching scheme (3) had the best performance for identifying true pairs of records from the same individual, with 69.8% sensitivity, 100% specificity, and strong agreement with true matches based on a kappa test of agreement (kappa = 0.8113). Additional matching schemes that considered diagnosis codes were investigated, but provided no improvements in performance. Linked pairs of matching entries were transformed to clusters of entries with common identifiers using the Transitive Record Linkage macro in SAS [35,36].

*Physician and pharmacy data source and management*

The Data Portal for physician and pharmacy information can be accessed at https://mqa-internet.doh.state.fl.us/downloadnet/GeneralInformation.aspx. Since license status prior to expiration is not listed for physicians with expired licenses, only physicians with a clear and active license at the time of data accession (01/2021) were included for further analysis. Therefore, 3.21% of practice addresses for physicians with unknown license status during the study period were excluded. Pharmacies with a non-expired license during the study period that were listed as closed at the time of data accession (03/2021) were included during the last full year prior to license expiration.
